# Effect of corneal stiffness decrease on axial length elongation in myopia determined based on a mathematical estimation model

**DOI:** 10.3389/fbioe.2023.1145032

**Published:** 2023-04-10

**Authors:** Qi Ren, Zhe Chu, Wei Cui, Lu Cheng, Wenjie Su, Hao Cheng, Jie Wu

**Affiliations:** ^1^ Eye Institute of Shandong First Medical University, Qingdao Eye Hospital of Shandong First Medical University, Qingdao, China; ^2^ State Key Laboratory Cultivation Base, Shandong Provincial Key Laboratory of Ophthalmology, Qingdao, China; ^3^ School of Ophthalmology, Shandong First Medical University, Qingdao, China; ^4^ State Key Laboratory of Ophthalmology, Zhongshan Ophthalmic Center, Sun Yat-sen University, Guangzhou, China; ^5^ Department of Ophthalmology, The First Affiliated Hospital of Guangzhou Medical University, Guangzhou, China

**Keywords:** spherical equivalent error, axial length, dynamic corneal response, stress-strain index, myopia

## Abstract

**Purpose:** To investigate the relationship between the corneal material stiffness parameter stress-strain index (SSI) and axial length (AL) elongation with varying severities of myopia, based on a mathematical estimation model.

**Methods:** This single-center, cross-sectional study included data from healthy subjects and patients preparing for refractive surgery in the Qingdao Eye Hospital of Shandong First Medical University. Data were collected from July 2021 to April 2022. First, we performed and tested an estimated AL model ( $AL_{Morgan}$ ) based on the mathematical equation proposed by Morgan. Second, we proposed an axial increment model ( ΔAL ) corresponding to spherical equivalent error (SER) based on ${AL}_{emmetropia}$ ( $AL_{Morgan}$ at SER = 0) and subject’s real AL. Finally, we evaluated the variations of ΔAL with SSI changes based on the mathematical estimation model.

**Results:** We found that AL was closely associated with $AL_{Morgan}$ (*r* = 0.91, *t* = 33.8, *p* < 0.001) with good consistency and SER was negatively associated with ΔAL (*r* = −0.89, *t* = −30.7, *p* < 0.001). The association of SSI with AL, ${AL}_{emmetropia}$ , and ΔAL can be summarized using the following equations: AL=27.7−2.04×SSI , ${AL}_{emmetropia}=23.2+0.561\times SSI$ , and ΔAL=4.52−2.6×SSI . In adjusted models, SSI was negatively associated with AL (Model 1: *β* = −2.01, *p* < 0.001) and ΔAL (Model 3: *β* = −2.49, *p* < 0.001) but positively associated with $AL_{emmetropia}$ (Model 2: *β* = 0.48, *p* < 0.05). In addition, SSI was negatively associated with ΔAL among subjects with AL ≥ 26 mm (*β* = −1.36, *p* = 0.02).

**Conclusion:** AL increased with decreasing SSI in myopia.

## 1 Introduction

Myopia is a refractive error in which light rays enter the eye parallel to the optic axis and are focused in front of the retina when ocular accommodation is relaxed. Usually, this results from a long eyeball, but it can also be caused by an overly curved cornea and/or lens with increased optical power (Flitcroft et al., 2019). As a general phenomenon of optometry, the nature of myopia and the relationship between myopia and refractive development remain unknown. However, it is certain that scleral remodeling plays a key role in both refractive development and myopia progression. Clinical and experimental studies on the biochemical and biomechanical properties of the sclera have shown that it participates in the regulation of axial elongation during refractive development through changes in fibers and extracellular stroma. The spherical equivalent error (SER) is normally distributed after birth, and the mean refractive error is hyperopic (Cook and Glasscock, 1951). Ideally, the sclera should stop elongating once the SER decrease reaches the endpoint of emmetropization, which represents the end of the refractive development period. However, in some individuals, the sclera continues to elongate until myopia develops. As stated earlier, there is no anatomical emmetropization endpoint between myopia and refractive development (Morgan et al., 2010) (Ma et al., 2021); even the normal refractive state is usually a dynamic equilibrium achieved by adjusting mild hyperopia through the lens and other refractive media (Anderson et al., 2010). There is insufficient evidence on the difference in scleral remodeling between non-high myopia and high myopia occurrence, especially high myopia with pathological changes. Likewise, axial myopia, a myopic refractive state attributed to excessive axial elongation, is closely related to scleral remodeling (Flitcroft et al., 2019); however, the differences in the mechanism of scleral remodeling between axial and non-axial myopia are unknown. Thus, there are differences between the biochemical and biomechanical properties of the scleral wall; therefore, understanding the mechanisms of biochemical and biomechanical differences in refractive development and varying severities of myopia is essential to determine the mechanism of myopia.

The refractive status of the cornea is another key factor in understanding myopia. Unlike sclera, the cornea develops by the age of 10 years, and axial elongation can last up to 20 years (Jones et al., 2005) (Wong et al., 2010). The mechanism of corneal biomechanical and behavioral changes related to myopia progression is unclear. One hypothesis is that the cornea undergoes similar changes in mechanical strength owing to scleral thinning and local dilation of the posterior sclera in highly myopic eyes (Rada et al., 2006). Previous reports have shown that axial length (AL) changes in myopia are associated with modifications of the corneal structure (Bueno-Gimeno et al., 2014). Therefore, studying the relationship between corneal biomechanical properties and AL elongation is key to understanding the pathogenesis of myopia and determining its prevention strategies.

Corneal biomechanical properties can be measured by assessing the cornea’s response to stress. Corneal stress can be achieved by applying an external force to the cornea by an air pulse; the ability of the cornea to resist deformation is known as stiffness, which depends on the elastic modulus and size and shape of the cross-section in the direction of force application (Roberts et al., 2016). Elastic modulus is a commonly used parameter for evaluating corneal stiffness (Kling and Hafezi, 2017). It is defined as the slope of the stress-strain curve of a material and describes the degree to which the load (stress) affects the deformation (strain) of the material under specific conditions. The higher the elastic modulus, the stiffer is the material. Because harder materials require greater force to deform (Roberts, 2014), the elastic modulus of the cornea reflects its ability to resist elastic deformation. *In vivo* measurements have shown that corneal stiffness decreases with increasing severities of myopia (Song et al., 2022) (Plakitsi et al., 2011) (Kang et al., 2018) (Han et al., 2020), indicating that corneal biomechanical changes are involved in the progression of myopia. The stress-strain index (SSI) is a corneal biomechanical parameter provided by Corvis ST Tonometry to evaluate the stiffness of corneal materials, which can intuitively and quantitatively reflect the stress-strain relationship in corneal materials (Eliasy et al., 2019). Previous research has confirmed that a decreased SSI is associated with high myopia based on SER (Liu et al., 2022). As a morphological variable related to axial myopia, theoretically, AL should correlate more with SSI than SER because AL elongation may be caused by a decrease in scleral stiffness. In fact, SSI at AL ≥ 26 mm was found to be smaller than that at AL < 26 mm but was not significantly correlated with AL in the respective groups (Liu et al., 2021) (Chu et al., 2022). To explore the reasons for this, we introduced the corneal radius of curvature (CR) in our previous work using the AL/CR ratio as the linkage variable of AL and SER; we found that AL/CR, which is more related to the severity of myopia, showed a significant negative correlation with SSI (Chu et al., 2022). According to the SSI proposal, SSI should not be correlated with the corneal geometry parameter CR (Eliasy et al., 2019). Therefore, we conjecture that AL increment that matches the CR change may be the driving force of the relationship between SSI and AL. For this reason, to investigate the relationship between SSI and AL elongation with varying severities of myopia, we developed and tested a mathematical model of AL increment corresponding to myopia progression using data from medical service records of a single center. This model is based on the existing mathematical approach for the theoretical estimation of AL in a cross-sectional study (Morgan et al., 2020).

## 2 Methods

### 2.1 Clinical data

We collected data for this study from July 2021 to April 2022 from the Picture Archiving and Communication System and Hospital Information System at the Qingdao Eye Hospital of Shandong First Medical University. The study population included healthy subjects and patients preparing for refractive surgery. Subjects were excluded if they met any of the following criteria: 1) 3 diopter (3D) power or more of astigmatism, 2) use of contact lenses, 3) history or suspicion of corneal diseases such as keratoconus, and 4) history of eye surgery.

For eligibility, the medical records of all subjects required the inclusion of a complete medical history and ophthalmic examinations on the same day, including comprehensive optometry results after mydriasis, AL (OA 2000, Tomey, Japan), anterior CR (as mean of Kflat and Ksteep) in a diameter range of 3 mm (OA 2000, Tomey, Japan), and the corneal biomechanical parameter SSI (Corvis ST, Oculus, Wetzlar, Germany). Only measurements with “OK” quality specifications were included in this analysis. All research procedures were conducted following the principles of the Declaration of Helsinki and were approved by the Ethics Committee of the Qingdao Eye Hospital of Shandong First Medical University.

### 2.2 Mathematical model

1) The theoretical estimation of AL was calculated using the mathematical model proposed by Morgan et al. (2020).

Morgan formula:

$$AL_{Morgan}=1/\left[\left(0.22273/CR\right)+0.00070\times SER+0.01368\right]$$

(CR = anterior corneal surface radius of curvature, as mean of Kflat and Ksteep; SER = spherical equivalent refractive error at the corneal plane (D). All SERs in this study were obtained from comprehensive optometric results after sufficient mydriasis).

2) The mathematical models of emmetropic AL, corresponding to corneal refraction, and AL increment of ametropia were calculated as:

$$AL_{emmetropia}=1/\left[\left(0.22273/CR\right)+0.01368\right]$$

$$\Delta AL=AL-AL_{emmetropia}$$

**FIGURE 1:**
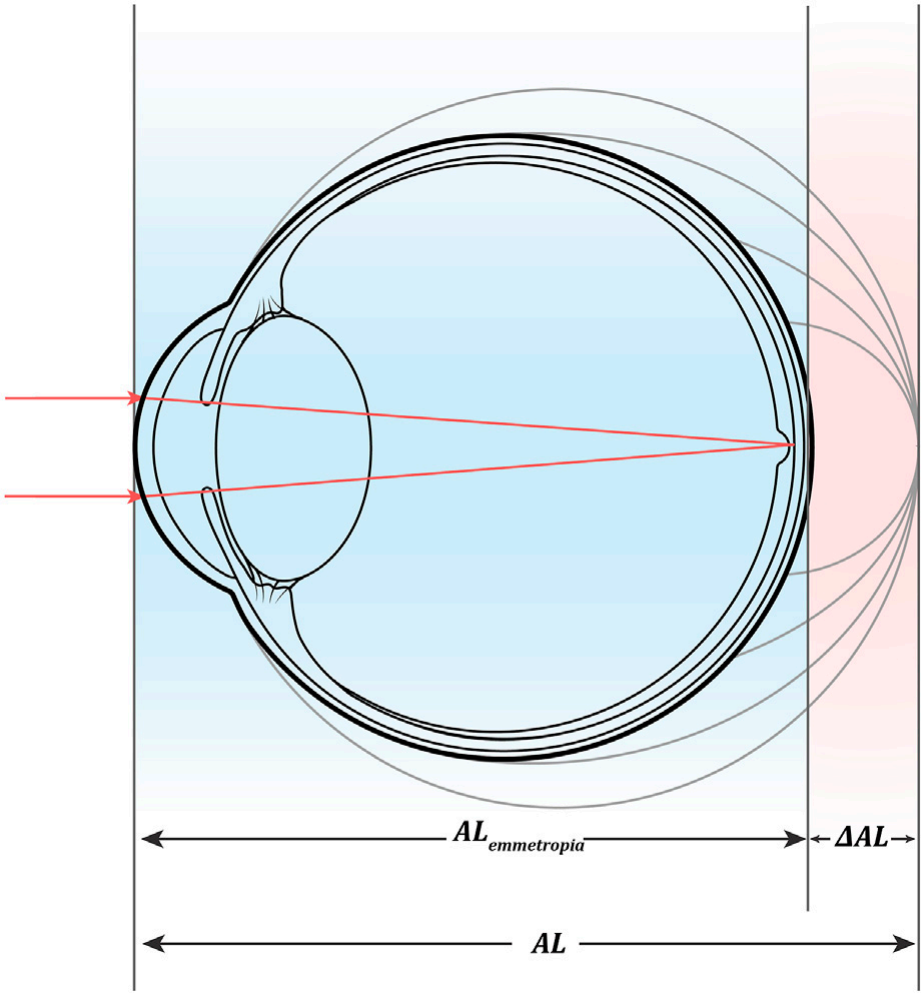
Mathematical model of axial increment.

$AL_{emmetropia}$ is the focal length of lens refraction, which represents emmetropia AL model that matches the actual lens power. ΔAL is the distance between imaging focus and photoreceptors. AL is the actual axial length (Figure 1).

### 2.3 Statistical analysis

Sociodemographic and clinical variables were summarized using descriptive statistics. The Pearson correlation test and Bland-Altman analysis were performed to analyze the relationship between SER and ΔAL . To examine the potential association of SSI with AL, $AL_{emmetropia}$ , and ΔAL , a series of linear regression models were conducted: model 1 with AL as the dependent variable and SSI as the independent variable was adjusted for age, gender, and CCT; model 2 with $AL_{emmetropia}$ as the dependent variable and SSI as the independent variable was adjusted for age, gender, and CCT; model 3 with ΔAL as the dependent variable and SSI as the independent variable was adjusted for age, gender, and CCT. To examine whether the association of SSI and ΔAL still exists in subjects with AL ≥ 26 mm, an additional linear regression model was performed by restricting our sample to individuals with AL ≥ 26 mm. Several key assumptions of linear regression, such as normality of residuals, homogeneity of variance, linearity, and independence, were checked. Our models did not violate these key assumptions. All data analyses were conducted using R statistical software version 4.2.2 (Team, 2014). The significance level was set at *p* < 0.05.

## 3 Results

### 3.1 Sample characteristics

As summarized in Table 1, our study included 267 subjects. In Table 1, values are expressed as mean (standard deviation) for continuous variables (i.e., age and AL) and sample size (percentage) for gender.

**TABLE 1:** Characteristics of our study sample.

Characteristic	N = 267^a^
Age, years	22 (8)
Gender	
Female	145 (54%)
Male	122 (46%)
SER (OD), diopter	−6.04 (3.07)
SER (OS), diopter	−5.71 (3.06)
SSI (OD)	0.82 (0.15)
SSI (OS)	0.84 (0.15)
CCT (OD), mm	544 (34)
CCT (OS), mm	544 (34)
CR (OD), mm	7.78 (0.24)
CR (OS), mm	7.76 (0.30)
AL (OD), mm	26.01 (1.48)
AL (OS), mm	25.88 (1.48)
$AL_{Morgan}$ (OD), mm	26.28 (1.40)
$AL_{Morgan}$ (OS), mm	26.13 (1.76)
$AL_{emmetropia}$ (OD), mm	23.64 (0.50)
$AL_{emmetropia}$ (OS), mm	23.59 (0.64)
ΔAL (OD), mm	2.38 (1.35)
ΔAL (OS), mm	2.29 (1.44)

Abbreviations: OD, for right eye; OS, for left eye; SER, spherical equivalent error; SSI, stress-strain index; CCT, central corneal thickness; CR, corneal curvature; AL, axial length.

^a^ n (%); Mean (SD).

### 3.2 Correlation between AL and $AL_{Morgan}$

**FIGURE 2:**
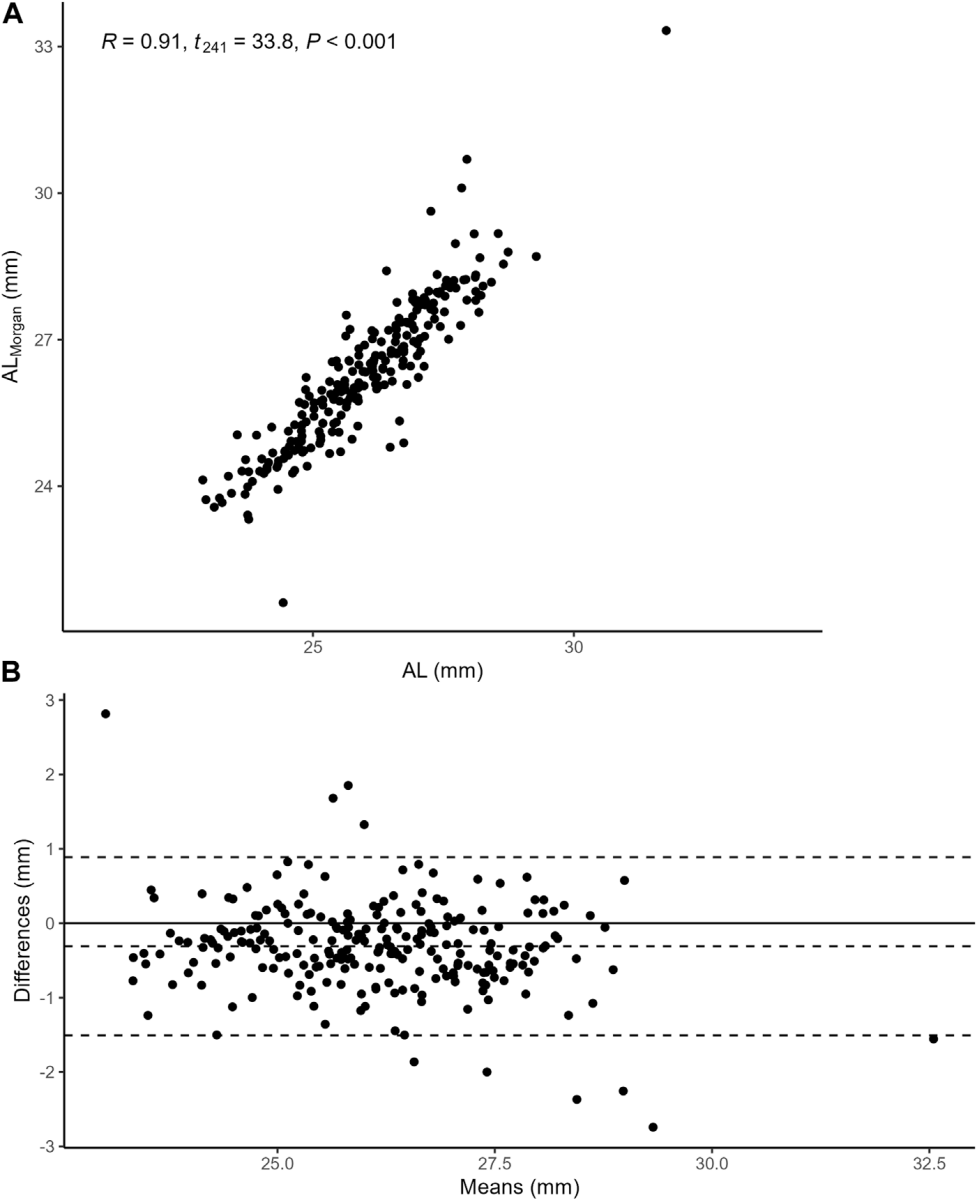
**(A)** the Pearson correlation between AL and $AL_{Morgan}$ . AL was closely associated with $AL_{Morgan}$ (*r* = 0.91, *t* = 33.8, *p* < 0.001). Abbreviation: AL, Axial length. **(B)** Bland-Altman analysis of AL and $AL_{Morgan}$ . The $AL_{Morgan}$ values are 0.31 ± 0.61 mm larger than the AL values, with 95% limits of agreement from −1.51 to 0.89 mm.

To examine the correlation between AL and $AL_{Morgan}$ , the Pearson correlation test and Bland-Altman analysis were performed. As displayed in Figures 2A, B, AL was closely associated with $AL_{Morgan}$ (*r* = 0.91, *t* = 33.8, *p* < 0.001) with good consistency.

### 3.3 AL, $AL_{emmetropia}$ , and ΔAL

**FIGURE 3:**
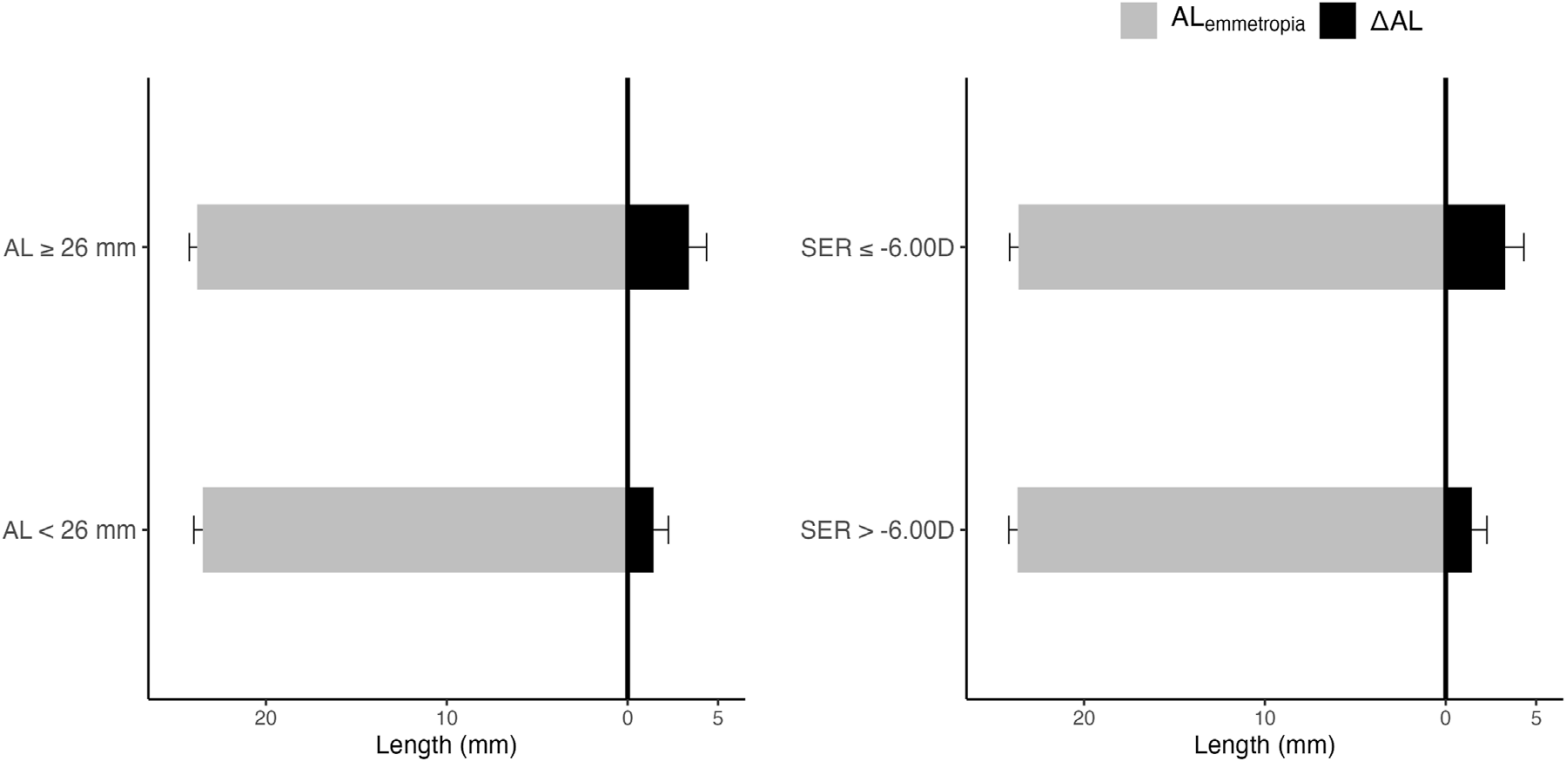
AL, $AL_{emmetropia}$ , and ΔAL . The left panel of Figure 3 shows levels of $AL_{emmetropia}$ and ΔAL in subjects with AL < 26 mm and AL ≥ 26 mm. The right panel of Figure 3 shows levels of $AL_{emmetropia}$ and ΔAL in subjects with SER > −6.00D and SER ≤ −6.00D. The grey area represents $AL_{emmetropia}$ . The black area represents ΔAL . Abbreviations: AL, Axial length; SER, Spherical equivalent error.

Figure 3 demonstrates the relationship between AL, $AL_{emmetropia}$ , and ΔAL . The left panel of Figure 3 shows levels of $AL_{emmetropia}$ and ΔAL in subjects with AL < 26 mm and AL ≥ 26 mm. The right panel of Figure 3 shows levels of $AL_{emmetropia}$ and ΔAL in subjects with SER > −6.00D and SER ≤ −6.00D.

### 3.4 Correlation between SER and ΔAL

**FIGURE 4:**
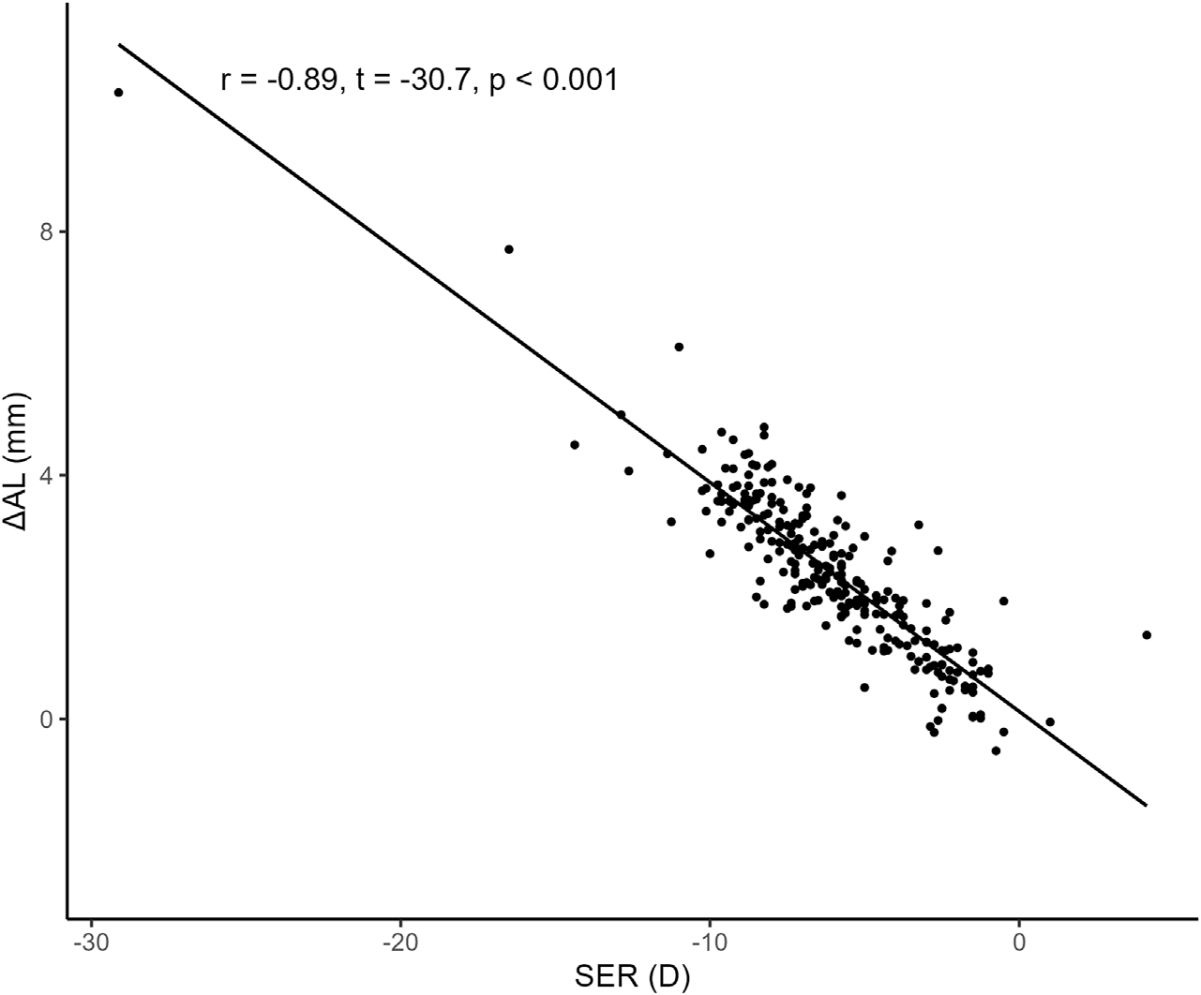
Correlation between SER and ΔAL . We found that SER was negatively associated with ΔAL (*r* = −0.89, *t* = −30.7, *p* < 0.001). Abbreviations: SER, Spherical equivalent error; AL, Axial length.

To examine the correlation between SER and ΔAL , the Pearson correlation test was performed. Figure 4 shows that SER was negatively associated with ΔAL (*r* = −0.89, *t* = −30.7, *p* < 0.001).

### 3.5 Summary of linear regression models

**FIGURE 5:**
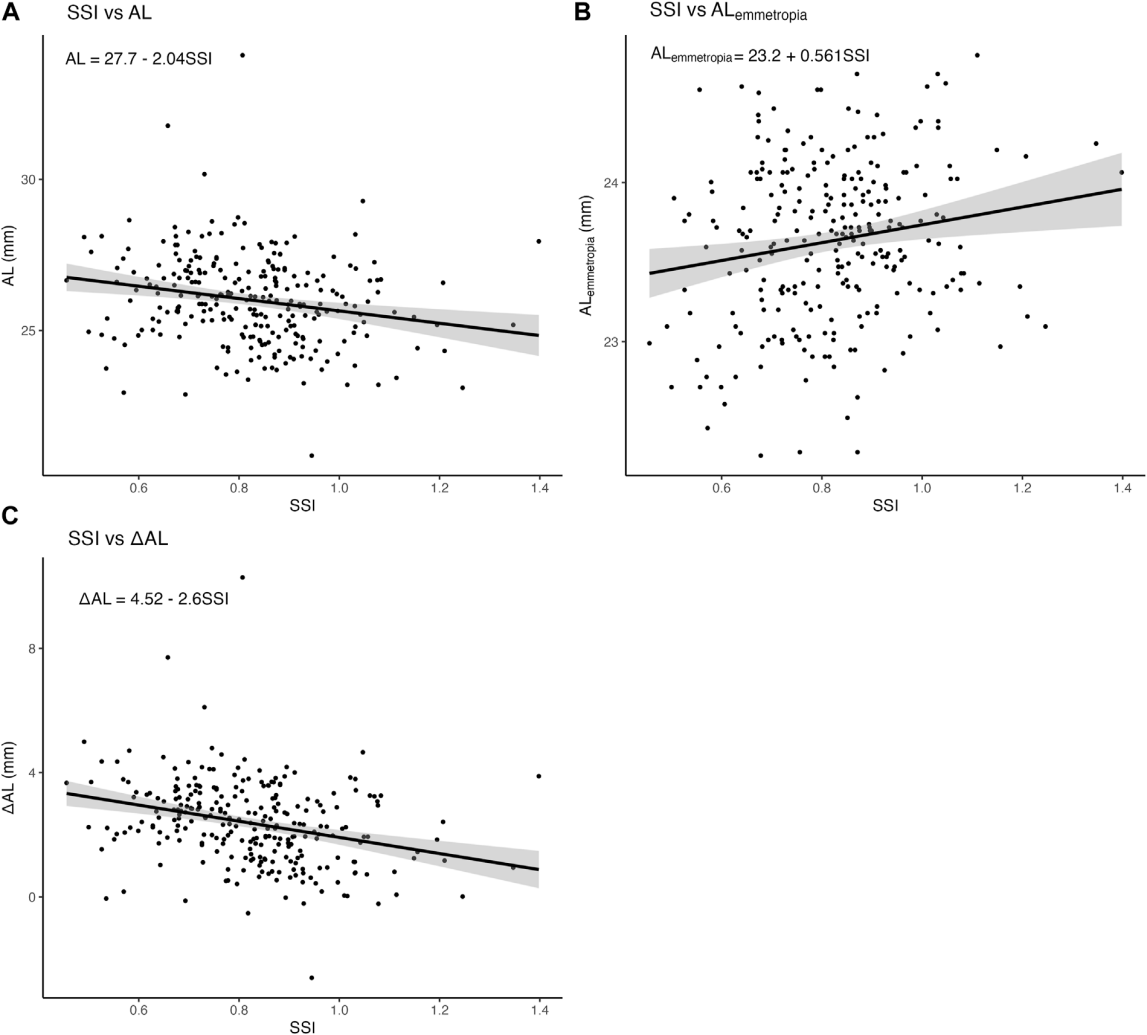
Associations of SSI with AL, $AL_{emmetropia}$ , and ΔAL . **(A)** shows the relationship between SSI and AL **(B)** shows the relationship between SSI and $AL_{emmetropia}$ **(C)** shows the relationship between SSI and ΔAL . Abbreviations: SSI, Stress-strain index; AL, Axial length.

First, we constructed three simple linear regression models to examine the associations of SSI with AL, ${AL}_{emmetropia}$ , and ΔAL . The statistical equations and scatter plots are demonstrated in Figure 5. The association of SSI with AL can be summarized using the following equation: AL=27.7−2.04×SSI . The association of SSI with ${AL}_{emmetropia}$ can be summarized using the following equation: ${AL}_{emmetropia}=23.2+0.561\times SSI$ . The association of SSI with ΔAL can be summarized using the following equation: ΔAL=4.52−2.6×SSI

**FIGURE 6:**
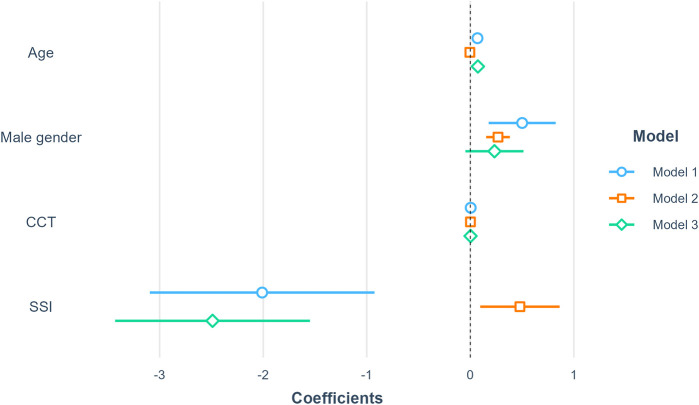
Summary of linear regression models. Model 1 included AL as the dependent variable; Model 2 included $AL_{emmetropia}$ as the dependent variable; Model 3 included ΔAL as the dependent variable. All coefficients were reported using unstandardized coefficients, and the 95% confidence intervals were used to describe the uncertainties of coefficients. Abbreviations: SSI, Stress-strain index; CCT, Central corneal thickness; AL, Axial length.

Second, several multiple linear regression models were conducted: model 1 with AL as the dependent variable and SSI as the independent variable was adjusted for age, gender, and CCT; model 2 with ${AL}_{emmetropia}$ as the dependent variable and SSI as the independent variable was adjusted for age, gender, and CCT; model 3 with ΔAL as the dependent variable and SSI as the independent variable was adjusted for age, gender, and CCT. These models are summarized in Table 2. In adjusted models, we found that SSI was negatively associated with AL (Model 1: *β* = −2.01, *p* < 0.001) and ΔAL (Model 3: *β* = −2.49, *p* < 0.001). However, SSI was positively associated with $AL_{emmetropia}$ (Model 2: *β* = 0.48, *p* < 0.05). A forest plot was also created to visualize the coefficients and corresponding 95% confidence intervals of the three models (Figure 6).

**TABLE 2:** Summary of linear regression models.

	Model 1	Model 2	Model 3
(Intercept)	23.84 ***	22.43 ***	1.40
	[21.09, 26.58]	[21.46, 23.40]	[−0.98, 3.79]
Age	0.07 ***	−0.00	0.07 ***
	[0.05, 0.09]	[−0.01, 0.00]	[0.06, 0.09]
Male gender	0.50 **	0.27 ***	0.23
	[0.18, 0.82]	[0.15, 0.38]	[−0.05, 0.51]
CCT	0.00	0.00	0.00
	[−0.00, 0.01]	[−0.00, 0.00]	[−0.00, 0.01]
SSI	−2.01 ***	0.48 *	−2.49 ***
	[−3.09, −0.93]	[0.10, 0.86]	[−3.43, −1.55]
N	267	267	267

***p < 0.001; **p < 0.01; *p < 0.05.

Model 1 included AL as the dependent variable; Model 2 included $AL_{emmetropia}$ as the dependent variable; Model 3 included ΔAL as the dependent variable. N represents the sample size in each model. The 95% confidence intervals were used to describe the uncertainties of coefficients. All coefficients were reported using unstandardized coefficients. Abbreviations: SSI, stress-strain index; CCT, central corneal thickness; AL, axial length.

**FIGURE 7:**
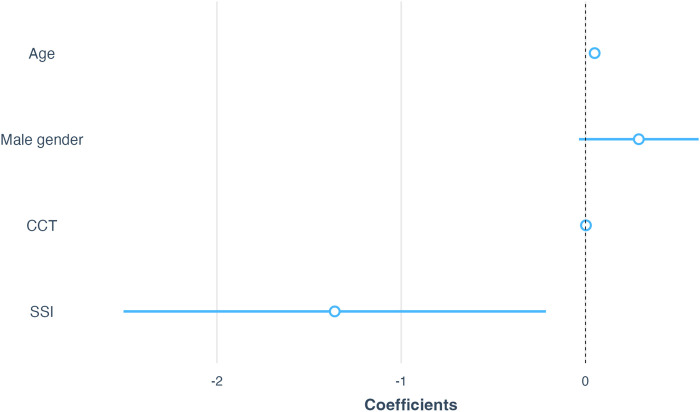
Association of SSI with ΔAL among subjects with AL ≥ 26 mm. All coefficients were reported using unstandardized coefficients, and the 95% confidence intervals were used to describe the uncertainties of coefficients. Abbreviations: SSI, Stress-strain index; CCT, Central corneal thickness; AL, Axial length.

To examine whether the association of SSI and ΔAL still exists in subjects with AL ≥ 26 mm, an additional linear regression model was performed by restricting our sample to individuals with AL ≥ 26 mm. We found that SSI was negatively associated with ΔAL among subjects with AL ≥ 26 mm (Figure 7; *β* = −1.36, *p* = 0.02).

## 4 Discussion

By analyzing the data of medical service records and the mathematical estimation model of AL, we found that the AL model of refractive error ( ΔAL ) based on the Morgan formula could be established accurately. SSI showed a negative correlation with AL increment, even for subjects with AL ≥ 26 mm.

AL changes are reflective of the regulatory processes of the sclera, especially those of the posterior sclera. The cornea and posterior sclerae are not directly connected structurally. Thus, the association between AL increase and SSI should be interpreted as an indirect association through the level of axial myopia, if the acquisition of SSI is not affected by the posterior eyeball structure, which means that corneal material stiffness decreases significantly with the deepening of axial myopia. However, SSI measured using Corvis ST is different from the elastic modulus of corneal tissue measured in the laboratory, as dynamic corneal responses may be affected by the internal structures and sclera stiffness (Kling and Marcos, 2013); to rule out this possibility, more targeted histological evidence is needed, although Corvis ST has good tissue accuracy and reproducibility in assessing biomechanical changes in dynamic corneal response. A recent theory of scleral hypoxia in the development of axial myopia suggests that myopic visual information disrupts retinal dopamine homeostasis and causes choroidal blood flow reduction and scleral hypoxia, resulting in scleral extracellular matrix remodeling and, eventually, scleral elongation (Zhao et al., 2020). Chronic hypoxemia can cause corneal stromal thinning (Bojarun et al., 2019) (Pang et al., 2021), and it is necessary to investigate whether the involvement of factors such as aqueous humor oxygen content and contact lens oxygen transmittance in reducing corneal biomechanical strength is associated with myopia progression (Liesegang, 2002).

An interesting finding was that our model’s intermediate quantity $AL_{emmetropia}$ , which represented the axial extension of the “non-myopic component” in the model, was positively correlated with SSI. As per the mathematical estimation model, ΔAL is responsible for the larger weight factor of the negative correlation between AL and SSI, which clarifies why the correlation between SSI and AL is not significant as compared to SER, especially as AL is extended (Liu et al., 2022) (Chu et al., 2022) (Liu et al., 2021). However, according to the $AL_{emmetropia}$ formula, SSI is correlated with $AL_{emmetropia}$ , which indicates that SSI is correlated with the corneal curvature of the front surface, and rationally, as a material index, SSI should be independent of corneal geometry to estimate the material stiffness as per the original publication (of SSI) (Eliasy et al., 2019). Before high myopia, the scleral wall expands nearly uniformly in all directions (Baohong et al., 2016). Therefore, we speculated that $AL_{emmetropia}$ may reflect the orbital volume or size of the eyeball at the emmetropization stage. However, as mentioned above, because of the dynamic balance between CR and AL, $AL_{emmetropia}$ cannot be equivalent to AL at the end of refractive development; therefore, it cannot be completely equivalent to the size of the eyeball. Thus, the relationship between SSI and $AL_{emmetropia}$ warrants further discussion.

Since the occurrence of SSI, many remarkable studies have reported on the relationship between corneal biomechanical behavior and myopia. This study is the first to apply mathematical modeling to assess the relationship between SSI and AL increment with myopia progression. The results clarify why SSI is more significantly related to SER than to AL and confirm the hypothesis that SSI is positively correlated with scleral elongation even at AL ≥ 26 mm. This finding supports the idea that the characteristics of the anterior segment of the eyeball change with increasing severity of myopia (Dhakal et al., 2020). Notably, the Morgan formula used to develop this mathematical model has been validated in a previous study (Queiros et al., 2022), highlighting the importance of the results of this study. However, our study has some limitations that should be considered. First, we used a mathematical model, and the databases did not contain information on the refractive power of other media, such as the aqueous humor, lens and vitreous, and accommodation, which could be used to estimate the distance between the imaging location and the photoreceptor. Thus, our model weights may be biased towards the refractive power of the corneal surface. However, if such a bias exists, it would not change our findings because we tested the ΔAL model with SER after mydriasis and established a suitable fit. Second, the sample size of the study was small; owing to the large variability in SSI measurements relative to the range of values, a larger sample size is needed to refine the study results. Finally, given the observational study design, the models may have had residual confounding effects, although we adjusted for several key variables.

Generally, structural changes during refractive development and myopia are interrelated. As observed in this study, any change in the morphological parameters of myopia, such as scleral dilation and corneal flattening, is associated with several other confounding changes, and it is difficult to analyze these changes separately and determine the causal relationship between them. Evaluating the biomechanical properties of the eyeball structure may aid in understanding the underlying causes of morphological changes with myopia; dilation of the posterior sclera is likely the result rather than the cause of sclera stiffness decrease in myopia (Sedaghat et al., 2020; Hon et al., 2017; Haseltine et al., 2012). Additionally, the lag in morphological changes may be avoided by analyzing the biomechanical properties of the eyeball structure in myopia.

In conclusion, ΔAL increased with decreasing SSI in myopia. In addition, the relationship between SSI and axial length was mainly driven by the relationship between SSI and AL increment, which was gradually masked by the positive correlation effect between SSI and $AL_{emmetropia}$ as AL increased.

## Data Availability

The original contributions presented in the study are included in the article/Supplementary Material, further inquiries can be directed to the corresponding author.

## References

[B1] Anderson H. A. Glasser A. Manny R. E. Stuebing K. K. (2010). Age-related changes in accommodative dynamics from preschool to adulthood. Invest. Ophthalmol. Vis. Sci. 51, 614–622. 10.1167/iovs.09-3653 19684002 PMC2869066

[B2] Baohong W. Ge Y. Jingliang C. Xuemin J. (2016). The three-dimensional MRI for estimating the shape of eyeball of emmetropia and myopia. Chin. J. Ocul. Traum Occupat Eye Dis., 413–416.

[B3] Bojarun A. Vieversyte Z. Jaruseviciene R. Galgauskas S. Asoklis R. Zablockis R. (2019). Effect of obstructive sleep apnea on corneal morphological characteristics. Cornea 38, 1576–1581. 10.1097/ico.0000000000002069 31356414

[B4] Bueno-Gimeno I. Espana-Gregori E. Gene-Sampedro A. Lanzagorta-Aresti A. Pinero-Llorens D. P. (2014). Relationship among corneal biomechanics, refractive error, and axial length. Optom. Vis. Sci. 91, 507–513. 10.1097/opx.0000000000000231 24705484

[B5] Chu Z. Ren Q. Chen M. Cheng L. Cheng H. Cui W. (2022). The relationship between axial length/corneal radius of curvature ratio and stress-strain index in myopic eyeballs: Using Corvis ST tonometry. Front. Bioeng. Biotechnol. 10, 939129. 10.3389/fbioe.2022.939129 36046672 PMC9420864

[B6] Cook R. C. Glasscock R. E. (1951). Refractive and ocular findings in the newborn. Am. J. Ophthalmol. 34, 1407–1413. 10.1016/0002-9394(51)90481-3 14877971

[B7] Dhakal R. Vupparaboina K. K. Verkicharla P. K. (2020). Anterior sclera undergoes thinning with increasing degree of myopia. Invest. Ophthalmol. Vis. Sci. 61, 6. 10.1167/iovs.61.4.6 PMC7401898 32271887

[B8] Eliasy A. Chen K. J. Vinciguerra R. Lopes B. T. Abass A. Vinciguerra P. (2019). Determination of corneal biomechanical behavior *in-vivo* for healthy eyes using CorVis ST tonometry: Stress-strain index. Front. Bioeng. Biotechnol. 7, 105. 10.3389/fbioe.2019.00105 31157217 PMC6532432

[B9] Flitcroft D. I. He M. Jonas J. B. Jong M. Naidoo K. Ohno-Matsui K. (2019). Imi - defining and classifying myopia: A proposed set of standards for clinical and epidemiologic studies. Invest. Ophthalmol. Vis. Sci. 60, M20–M30. 10.1167/iovs.18-25957 30817826 PMC6735818

[B10] Han F. Li M. Wei P. Ma J. Jhanji V. Wang Y. (2020). Effect of biomechanical properties on myopia: A study of new corneal biomechanical parameters. BMC Ophthalmol. 20, 459. 10.1186/s12886-020-01729-x 33213408 PMC7678063

[B11] Haseltine S. J. Pae J. Ehrlich J. R. Shammas M. Radcliffe N. M. (2012). Variation in corneal hysteresis and central corneal thickness among black, hispanic and white subjects. Acta Ophthalmol. 90, e626–e631. 10.1111/j.1755-3768.2012.02509.x 22938724

[B12] Hon Y. Chen G. Z. Lu S. H. Lam D. C. Lam A. K. (2017). High myopes have lower normalised corneal tangent moduli (less 'stiff' corneas) than low myopes. Ophthalmic Physiol. Opt. 37, 42–50. 10.1111/opo.12335 27873338

[B13] Jones L. A. Mitchell G. L. Mutti D. O. Hayes J. R. Moeschberger M. L. Zadnik K. (2005). Comparison of ocular component growth curves among refractive error groups in children. Invest. Ophthalmol. Vis. Sci. 46, 2317–2327. 10.1167/iovs.04-0945 15980217

[B14] Kang B. S. Wang L. K. Zheng Y. P. Guggenheim J. A. Stell W. K. Kee C. S. (2018). High myopia induced by form deprivation is associated with altered corneal biomechanical properties in chicks. PLoS One 13, e0207189. 10.1371/journal.pone.0207189 30419001 PMC6231665

[B15] Kling S. Hafezi F. (2017). Corneal biomechanics - a review. Ophthalmic Physiol. Opt. 37, 240–252. 10.1111/opo.12345 28125860

[B16] Kling S. Marcos S. (2013). Contributing factors to corneal deformation in air puff measurements. Invest. Ophthalmol. Vis. Sci. 54, 5078–5085. 10.1167/iovs.13-12509 23821200

[B17] Liesegang T. J. (2002). Physiologic changes of the cornea with contact lens wear. CLAO J. 28, 12–27. 11838985

[B18] Liu G. Rong H. Zhang P. Xue Y. Du B. Wang B. (2021). The effect of axial length elongation on corneal biomechanical property. Front. Bioeng. Biotechnol. 9, 777239. 10.3389/fbioe.2021.777239 34926423 PMC8677453

[B19] Liu Y. Pang C. Ming S. Fan Q. (2022). Effect of myopia and astigmatism deepening on the corneal biomechanical parameter stress-strain index in individuals of Chinese ethnicity. Front. Bioeng. Biotechnol. 10, 1018653. 10.3389/fbioe.2022.1018653 36420440 PMC9676639

[B20] Ma Y. Lin S. Morgan I. G. Rozema J. J. Iribarren R. Zhu J. (2021). Eyes grow towards mild hyperopia rather than emmetropia in Chinese preschool children. Acta Ophthalmol. 99, e1274–e1280. 10.1111/aos.14810 33942521 PMC9541634

[B21] Morgan I. G. Rose K. A. Ellwein L. B., and Refractive error study in children survey, G. (2010). is emmetropia the natural endpoint for human refractive development? An analysis of population-based data from the refractive error study in children (RESC). Acta Ophthalmol. 88, 877–884. 10.1111/j.1755-3768.2009.01800.x PMC2891782 19958289

[B22] Morgan P. B. Mccullough S. J. Saunders K. J. (2020). Estimation of ocular axial length from conventional optometric measures. Cont. Lens Anterior Eye 43, 18–20. 10.1016/j.clae.2019.11.005 31786071

[B23] Pang K. Lennikov A. Yang M. (2021). Hypoxia adaptation in the cornea: Current animal models and underlying mechanisms. Anim. Model Exp. Med. 4, 300–310. 10.1002/ame2.12192 PMC8690994 34977481

[B24] Plakitsi A. O'donnell C. Miranda M. A. Charman W. N. Radhakrishnan H. (2011). Corneal biomechanical properties measured with the Ocular Response Analyser in a myopic population. Ophthalmic Physiol. Opt. 31, 404–412. 10.1111/j.1475-1313.2011.00852.x 21615446

[B25] Queiros A. Amorim-De-Sousa A. Fernandes P. Ribeiro-Queiros M. S. Villa-Collar C. Gonzalez-Meijome J. M. (2022). Mathematical estimation of axial length increment in the control of myopia progression. J. Clin. Med. 11, 6200. 10.3390/jcm11206200 36294521 PMC9604591

[B26] Rada J. A. Shelton S. Norton T. T. (2006). The sclera and myopia. Exp. Eye Res. 82, 185–200. 10.1016/j.exer.2005.08.009 16202407

[B27] Roberts C. J. (2014). Concepts and misconceptions in corneal biomechanics. J. Cataract. Refract Surg. 40, 862–869. 10.1016/j.jcrs.2014.04.019 24857435

[B28] Roberts C. J. Liu J. Ebook Central Academic C. (2016). Corneal biomechanics: From theory to practice. Amsterdam: Kugler Publications.

[B29] Sedaghat M. R. Momeni-Moghaddam H. Azimi A. Fakhimi Z. Ziaei M. Danesh Z. (2020). Corneal biomechanical properties in varying severities of myopia. Front. Bioeng. Biotechnol. 8, 595330. 10.3389/fbioe.2020.595330 33553113 PMC7859342

[B30] Song Y. Wu D. Shen M. Wang L. Wang C. Cai Y. (2022). Measuring human corneal stromal biomechanical properties using tensile testing combined with optical coherence tomography. Front. Bioeng. Biotechnol. 10, 882392. 10.3389/fbioe.2022.882392 35669060 PMC9163803

[B31] Team R. C. (2014). R: A language and environment for statistical computing. MSOR Connect. 1.

[B32] Wong H. B. Machin D. Tan S. B. Wong T. Y. Saw S. M. (2010). Ocular component growth curves among Singaporean children with different refractive error status. Invest. Ophthalmol. Vis. Sci. 51, 1341–1347. 10.1167/iovs.09-3431 19875656

[B33] Zhao F. Zhang D. Zhou Q. Zhao F. He M. Yang Z. (2020). Scleral HIF-1α is a prominent regulatory candidate for genetic and environmental interactions in human myopia pathogenesis. EBioMedicine 57, 102878. 10.1016/j.ebiom.2020.102878 32652319 PMC7348000

